# The Association between Vitamin D and Nonalcoholic Fatty Liver Disease Assessed by Controlled Attenuation Parameter

**DOI:** 10.3390/jcm10122611

**Published:** 2021-06-13

**Authors:** Nam Ju Heo, Hyo Eun Park, Ji Won Yoon, Min-Sun Kwak, Jong In Yang, Su Jin Chung, Jeong Yoon Yim, Goh Eun Chung

**Affiliations:** 1 Department of Nephrology, Internal Medicine, Seoul National University Hospital Healthcare System Gangnam Center, Seoul 06236, Korea; julie082007@naver.com; 2 Department of Cardiology, Internal Medicine, Seoul National University Hospital Healthcare System Gangnam Center, Seoul 06236, Korea; hyoeunmd1@gmail.com; 3 Department of Endocrinology, Internal Medicine, Seoul National University Hospital Healthcare System Gangnam Center, Seoul 06236, Korea; jwyoonmd@gmail.com; 4 Department of Gastroenterology and Hepatology, Internal Medicine, Seoul National University Hospital Healthcare System Gangnam Center, Seoul 03080, Korea; kms39@snuh.org (M.-S.K.); drmirinae@snuh.org (J.I.Y.); medjsj@snuh.org (S.J.C.); yjy@snuh.org (J.Y.Y.)

**Keywords:** vitamin D, hepatic steatosis, CAP

## Abstract

Background: An association between nonalcoholic fatty liver disease (NAFLD) and low vitamin D levels has been suggested. We investigated the relationship between vitamin D and NAFLD assessed by controlled attenuation parameter (CAP). Methods: We conducted a retrospective cohort study of apparently healthy subjects who underwent Fibroscan during health screening tests. NAFLD was diagnosed using CAP values. Results: Among the 1202 subjects (mean age 57.2 years, 60.6% male), 630 (52.4%) subjects had NAFLD with CAP ≥ 248 dB/m. Multivariable analysis was conducted after adjusting for metabolic risk factors including diabetes, hypertension, hypercholesterolemia, body mass index, high-density lipoprotein cholesterol, triglyceride and smoking. Higher vitamin D levels showed a lower risk of NAFLD compared to the lowest quartile of vitamin D in a dose-dependent manner (OR 0.68, 95% CI 0.47–1.00 in Q2 vs. Q1; OR 0.65, 95% CI 0.44–0.94 in Q3 vs. Q1; and OR 0.64, 95% CI 0.44–0.94 in Q4 vs. Q1). The highest quartile of vitamin D showed a decreased risk of a severe grade of steatosis (CAP ≥ 302 dB/m) compared to the lowest quartile (OR 0.52, 95% CI 0.31–0.87 in Q4 vs. Q1). Conclusions: Higher levels of serum vitamin D were associated with a decreased risk of CAP-defined NAFLD, compared to low levels of serum vitamin D. The association between NAFLD and vitamin D suggests that vitamin D may exert a protective role against NAFLD.

## 1. Introduction

Non-alcoholic fatty liver disease (NAFLD) is the most common liver disease, with a prevalence of 25% globally and 27% in Asia [1]. NAFLD is closely related to various metabolic conditions, such as visceral obesity, type 2 diabetes and cardiovascular disease, and has been regarded as a hepatic manifestation of metabolic syndrome [2]. Recently, the new definition as ‘metabolic (dysfunction)-associated fatty liver disease (MAFLD)’ has been introduced, and there is an emphasis on the role of metabolic dysfunction on the clinical outcome of patients with fatty liver disease [3]. Although liver biopsy is the gold standard for diagnosis and quantitation of fatty liver, its use in clinical practice, especially in asymptomatic subjects without overt liver disease, is extremely limited due to its invasiveness and possible sampling error. Therefore, ultrasonography is recommended as the first-line modality in clinical practices [4]. In addition to imaging modalities, a controlled attenuation parameter (CAP) during transient elastography using FibroScan^®^ has high sensitivity in detecting low-grade steatosis and a good correlation with grades of steatosis. Thus, CAP can be used to assess hepatic steatosis while enabling early and noninvasive detection of NAFLD at the subclinical stage [5,6,7].

Vitamin D is a sterol derivative synthesized by ultraviolet radiation from the skin and can also be obtained from the diet or food supplements. As a fat-soluble vitamin, it affects bone metabolism and immune function [8]. Vitamin D deficiency, characterized by low serum levels of 25-hydroxyvitamin D, is associated with the increased risk of various metabolic diseases, including metabolic syndrome [9,10], coronary artery disease [11] and chronic liver disease [8,12]. The association between vitamin D deficiency and NAFLD has been examined in previous studies. A lower vitamin D was an independent risk factor for NAFLD [13,14] and a predictor of the severity of NAFLD [15]. Rhee et al. reported that participants with higher vitamin D showed a significantly reduced risk for NAFLD [16]. A recent meta-analysis displayed a negative association between serum vitamin D levels and NAFLD, with a lower odds ratio (OR) = 0.64 [17]. However, several studies reported that serum vitamin D might not be associated with the histologic severity of NAFLD [18,19].

Based on a study of a health check-up population, we evaluated the association between vitamin D and NAFLD/MAFLD, defined by CAP using FibroScan^®^, which gives an objective value for early diagnosis of hepatic steatosis and fibrosis.

## 2. Methods

### 2.1. Study Population

This retrospective observational study included subjects who underwent routine health check-ups at the Seoul National University Hospital Healthcare System Gangnam Center between January 2018 and December 2020. The subjects underwent general health check-ups on a voluntary basis or through employer-sponsored coverage. The subjects were mostly free of symptoms and willingly received examinations including, FibroScan^®^ (Echosens, Paris, France) and blood samplings on the same day. Initially, a total of 1417 subjects were enrolled. For NAFLD, subjects who displayed any potential cause of chronic liver disease were excluded; 36 were positive for the hepatitis B virus, 12 were positive for the hepatitis C virus, and 167 had significant alcohol intake (>20 g/day for males and >10 g/day for females) [20]. As a result, 1202 subjects were included in the NAFLD analysis. For MAFLD, 109 subjects with missing information were excluded. The diagnosis of MAFLD was based on the previous criteria [3].

The study protocol adhered to the guidelines of the Declaration of Helsinki of 1975, as revised in 1983. The protocol was approved by the Institutional Review Board of Seoul National University Hospital (No. 2006-024-1130). Informed consent was waived by the board as researchers accessed and analyzed only de-identified data.

### 2.2. Measurement of Anthropometric and Laboratory Parameters

The methods employed in this study have been previously described in detail [21]. Anthropometric and laboratory parameters were taken on the same day of the health check-ups. Body weight and height were measured using a digital scale, and body mass index (BMI) was calculated by dividing weight (kg) by the squared value of height (m^2^). Well-trained personnel used a measuring tape to measure the waist circumference at the midpoint between the lower costal margin and anterior superior iliac crest.

Data regarding past medical history, comorbidities and medication history were obtained through subject-recorded questionnaires. Each subject was categorized as a smoker or non-smoker, and the amount of alcohol each patient consumed was calculated. Blood pressure was measured at least twice, and mean values of the measurements were recorded. Hypertension was defined as blood pressure ≥140/90 mmHg or receiving antihypertensive medications. Diabetes was defined as fasting blood glucose ≥126 mg/dL or receiving glucose-lowering agents. Subjects taking lipid-lowering agents were categorized as having hypercholesterolemia [21]. All blood samples were collected after a 12-h overnight fast. Laboratory tests included serum total cholesterol, triglycerides, high-density lipoprotein (HDL) cholesterol, fasting glucose, creatinine and high-sensitivity C-reactive protein (HS-CRP). Standard laboratory methods were used to perform all of these tests.

### 2.3. Measurement of Serum Vitamin D Levels

Serum levels of 25-hydroxyvitamin (OH) D_3_ were measured using a chemiluminescence immunoassay kit (Diasorin, Stillwater, OK, USA). Subjects were categorized as having either vitamin D deficiency (<20 ng/mL) or vitamin D sufficiency (≥20 ng/mL) [22,23]. Additionally, quartile 1 of serum vitamin D level was ≤14.6 ng/mL, quartile 2 was 14.7–19.5 ng/mL, quartile 3 was 19.6–26.7 ng/mL, and quartile 4 was ≥26.8 ng/mL. We used the lowest quartile (≤14.4 ng/mL) of vitamin D levels as a reference group.

### 2.4. Measurement of NAFLD Using CAP and Liver Stiffness

CAP and liver stiffness measurements (LSM) were obtained by FibroScan^®^ using an M or XL probe (Echosens, Paris, France). The procedure was performed by an experienced investigator who was blinded to the patients’ clinical information. The procedure was performed as described briefly [24]. The patient was positioned in dorsal decubitus with the right arm maximally abducted. FibroScan^®^ was performed on the right lobe through the intercostal spaces. The median LSM values were expressed in kilopascals (kPa), and the median CAP was expressed in dB/m. LSM values were considered reliable if 10 valid measurements were obtained and the interquartile range/median of the measurements were <0.3 or when the LS median was <7.1 kPa [25]. All of the patients with 10 valid shots were included in the analysis. In this study, CAP values of 248 and 302 dB/m were used to define NAFLD and grade the steatosis [7,26].

### 2.5. Statistical Analysis

Continuous variables were expressed as mean ± SD for normally distributed continuous variables, and categorical variables were expressed in number and percentage. To test for normality, the Kolmogorov-Smirnov test and the normal Q-Q plots were used. For non-normally distributed variables, log transformations were performed. Comparisons between normal and low vitamin D groups were performed using Student’s t-test for continuous variables and the chi-square test or Fisher’s exact test for categorical variables. To evaluate the parameters that affect NAFLD, univariate and multivariate logistic regression analyses were performed. Model 1 was adjusted for age, sex, hypertension, diabetes, hypercholesterolemia and BMI. Model 2 was further adjusted for HDL-cholesterol, triglyceride and smoking. All statistical analyses were performed using SPSS 22.0 (SPSS Inc., Chicago, IL, USA), and *p* values < 0.05 were considered statistically significant.

## 3. Results

### 3.1. Clinical Characteristics of Study Population 

The mean age of our study population was 57.2 years, and 60.6% of the subjects were male. Among the 1202 subjects, 614 (51.1%) displayed vitamin D deficiency. Clinical characteristics according to vitamin D level are summarized in Table 1. Compared with those with vitamin D sufficiency, individuals with vitamin D deficiency were younger, more frequently obese (BMI ≥ 25), current smokers and had a higher waist circumference (*p* < 0.05). Serum levels of total cholesterol and triglyceride were higher, while serum levels of HDL-cholesterol were lower in individuals with vitamin D deficiency than those with vitamin D sufficiency. Subjects with vitamin D deficiency had a higher prevalence of NAFLD for both CAP ≥ 248 and ≥ 302 dB/m (*p* < 0.05). The tertiles of LSM value were not different between the two groups.

### 3.2. Parameters Associated with NAFLD

Table 2 shows the association of each parameter with NAFLD defined by CAP ≥ 248 dB/m using univariate logistic regression analysis. Male sex, hypertension, diabetes, hypercholesterolemia, smoking, BMI, fasting glucose, triglyceride and HDL-cholesterol were significantly associated with NAFLD (*p* < 0.05). The serum vitamin D levels (≥ 20 (vs. <20 ng/mL)) were inversely associated with NAFLD defined as CAP ≥ 248 dB/m (Odds ratio (OR) 0.75, 95% confidence interval (CI) 0.60–0.95). When evaluated in quartiles, the highest quartile of serum vitamin D was associated with a lower risk of NAFLD compared to the lowest quartile (OR 0.60, 95% CI 0.44–0.83 in Q4 vs. Q1).

### 3.3. Association between NAFLD/MAFLD and Vitamin D Level

Multivariate logistic regression analysis was performed to evaluate the adjusted association between hepatic steatosis and vitamin D levels. When adjusting for age and sex, individuals with vitamin D sufficiency was associated with decreased risk of NAFLD defined as CAP ≥ 248 dB/m compared to those with vitamin D deficiency (OR 0.72, 95% CI 0.57–0.91). Upon further adjustments of multiple confounding factors including hypertension, diabetes, hypercholesterolemia, BMI, HDL-cholesterol triglyceride and smoking, individuals with vitamin D sufficiency was associated with a lower risk of NAFLD compared to those with vitamin D deficiency without statistical significance (OR 0.78, 95% CI 0.60–1.02, Table 3). When we evaluated vitamin D levels in quartiles, the highest quartile of vitamin D showed a dose-dependent relationship with NAFLD compared to the lowest quartile after adjusting multiple confounding factors (OR 0.68, 95% CI 0.47–1.00 in Q2 vs. Q1; OR 0.65, 95% CI 0.44–0.94 in Q3 vs. Q1 and OR 0.64; 95% CI 0.44–0.94 in Q4 vs. Q1). Among the eligible 1308 subjects, 44% showed MAFLD, and when we investigated the association between MAFLD and vitamin D level, although the significance attenuated, the results are similar to that of NAFLD (Table 4).

### 3.4. Association between Grade of Steatosis and Vitamin D Level

**Figure 1:**
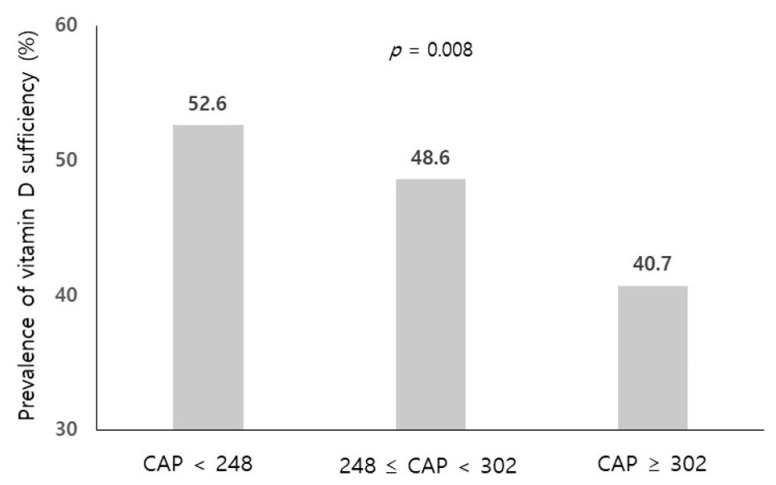
The prevalence of vitamin D sufficiency (≥20 ng/mL) according to the grade of hepatic steatosis. Compared using chi-square test. CAP, controlled attenuation parameter.

To evaluate the association between the grade of hepatic steatosis and vitamin D levels, we graded hepatic steatosis according to the CAP values (<248, 248–302 and ≥302 dB/m). The prevalence of vitamin D sufficiency (≥20 ng/mL) was significantly lower in the group with a severe grade of steatosis (CAP ≥ 302 dB/m) compared to the group with CAP > 248 dB/m (*p* = 0.008, Figure 1). When we performed multivariate ordinary regression analysis, the highest quartile of vitamin D showed a decreased risk of a severe grade of steatosis (CAP ≥ 302 dB/m) compared to the lowest quartile after adjusting multiple confounding factors (OR 0.52, 95% CI 0.31–0.87 in Q4 vs. Q1, Table 5).

## 4. Discussion

To the best of our knowledge, this is the first study to reveal a decreased risk of CAP-defined NAFLD in subjects with higher vitamin D compared to those with lower vitamin D. Moreover, there was a dose-response relationship between the grade of hepatic steatosis and vitamin D levels.

Several studies have shown that the prevalence of vitamin D deficiency in patients with NAFLD is higher than in healthy controls [15,27]. Recently, NAFLD has been renamed as metabolic dysfunction-associated fatty liver disease, and its association with vitamin D has been investigated [28]. In their study, NAFLD was usually diagnosed with abdominal ultrasonography. Although ultrasonography is a good modality to detect moderate–severe fatty liver, ultrasonography sensitivity decreases as the hepatic fatty infiltration decreases. In the presence of liver fat content below 20%, ultrasonography sensitivity was only 55% [4]. In addition, there are limitations to the reliability of such radiologic evaluations, and future studies should include reliability data such as inter- and intra-observer variations [29]. CAP with FibroScan^®^ has recently been introduced as a noninvasive method that detects and quantifies hepatic steatosis as well as fibrosis simultaneously with adequate sensitivity [26]. A previous study has shown that CAP values are significantly associated with the severity of ultrasonography-diagnosed hepatic steatosis, suggesting that increased CAP could be an early indicator of NAFLD [6]. Moreover, the usefulness of CAP is not limited to the liver. The association between CAP-defined NAFLD and increased arterial stiffness [30], the presence of coronary artery plaques [21] and gastroesophageal reflux disease [31] has been reported, suggesting the role of CAP as risk stratification for extrahepatic manifestations in patients with NAFLD.

Although there has been no consensus on how to define vitamin D deficiency, the cut points of total serum 25 (OH) D levels as 20 or 30 ng/mL were mostly used in previous studies [32,33]. However, the median vitamin D concentration varied among previous studies [18], and Rhee et al. has reported that the prevalence of vitamin D insufficiency was relatively high in Korean adults with a rate of more than 60% [34]. Therefore, additional categories of vitamin D deficiency or insufficiency are needed regarding the Korean population. This study showed no statistical significance regarding the association of NAFLD and vitamin D levels when the subjects were classified into two vitamin D categories. However, when the vitamin D levels were divided into quartiles, the significant relationship between NAFLD and vitamin D remained in all quartile levels. The tertiles of LSM value and surrogate marker of hepatic fibrosis were not different between the normal and low vitamin D groups in this study. The LSM values of most subjects were within the normal range as the subjects for health check-ups were composed of apparently healthy persons without overt liver disease.

The possible mechanisms linking NAFLD and low vitamin D have not been clearly elucidated. First, vitamin D and liver inflammation: since vitamin D has anti-inflammatory activity, a lower vitamin D level might cause dysfunction of adipose tissue and subsequent chronic inflammation, which may result in the development of NAFLD [35]. In the animal model, rats fed with vitamin D depletion showed greater hepatic steatosis and inflammation compared to the control group [36]. Second, vitamin D and insulin sensitivity: vitamin D insufficiency has been independently associated with metabolic syndrome [37] and insulin resistance [38], which is the key pathophysiology of NAFLD and supplementation of vitamin D has resulted in improving insulin sensitivity [39]. Della Pirgon et al. reported that low vitamin D level was associated with insulin resistance in obese NAFLD patients [40]. Third, it has been reported that vitamin D decreased the activation of hepatic stellate cells, suggesting a potential role to protect against liver fibrosis [41]. However, further studies evaluating the pathogenesis between NAFLD and vitamin D are necessary.

## 5. Limitations

Our study has some limitations. First, its cross-sectional design limits the ability to verify causality. Thus, we could not infer causal relationships from this study. Second, NAFLD was not biopsy-proven in this study. However, CAP measurement constitutes a good non-invasive biomarker of hepatic fat or fatty liver [7], although not in NASH and grading of hepatic steatosis. However, the cut-off values used in this study for grading hepatic steatosis were similar to those reported in the study based on the Korean population [42]. Third, we did not consider seasonal variations of vitamin D levels, sun exposure time and calcium or vitamin D supplement use in this study. Lastly, our study population of those who underwent health evaluations upon their own initiative may not represent the majority of the general Korean population, and this may contribute to selection bias.

## 6. Conclusions

There was a decreased risk of CAP-defined NAFLD in subjects with higher vitamin D compared to those with lower vitamin D in a dose-dependent manner. The association between NAFLD and vitamin D suggests that vitamin D may exert protective roles against NAFLD.

## Figures and Tables

**Table 1:** A comparison of baseline characteristics according to vitamin D level.

	Vitamin D Sufficiency (≥20 ng/mL) (*n* = 588)	Vitamin D Deficiency (<20 ng/mL) (*n* = 614)	*p*-Value
Age (years)	59.0 ± 10.3	55.5 ± 10.8	<0.001
Male, *n* (%)	355 (60.4)	374 (60.9)	0.849
Current smoking, *n* (%)	94 (16.0)	136 (22.1)	0.007
BMI (kg/m^2^)	24.0 ± 3.3	24.7 ± 3.6	0.707
BMI ≥ 25 (kg/m^2^)	207 (35.2)	262 (42.7)	0.008
Waist circumference (cm)	87.7 ± 9.2	88.9 ± 9.9	0.036
Systolic blood pressure, mmHg	122.1 ± 16.3	122.1 ± 16.5	0.972
Diastolic blood pressure, mmHg	80.4 ± 10.7	81.3 ± 10.9	0.149
Comorbidities			
Diabetes mellitus, *n* (%)	110 (18.7)	102 (16.6)	0.341
Hypertension, *n* (%)	183 (31.1)	169 (27.5)	0.171
Hypercholesterolemia, *n* (%)	190 (32.3)	145 (23.5)	0.002
Laboratory parameters			
Total cholesterol (mg/dL)	188.5 ± 40.1	194.5 ± 40.9	0.010
Triglyceride (mg/dL) ^+^	97 (68–137)	97 (70–149)	0.041
HDL-cholesterol (mg/dL)	55.8 ± 15.1	53.9 ± 15.0	0.031
Fasting glucose (mg/dL)	104.8 ± 22.1	105.9 ± 24.4	0.412
Creatinine (mg/dL)	0.85 ± 0.2	0.84 ± 0.2	0.786
HS-CRP (mg/dL)	0.13 ± 0.3	0.15 ± 0.4	0.297
Transient elastography			
Controlled attenuation parameter, dB/m ^+^	246 (215–285)	258 (217–300)	0.005
CAP ≥ 248	287 (48.8)	343 (55.9)	0.014
CAP ≥ 302	99 (16.8)	144 (23.5)	0.004
Liver stiffness measurement, kPa ^+^	3.6 (3.1–4.4)	4.2 (3.5–4.8)	0.851
Tertile 1 (<3.4)	195 (33.2)	194 (31.6)	0.359
Tertile 2 (3.4–4.2)	208 (35.4)	203 (33.1)	
Tertile 3 (≥4.3)	185 (31.5)	217 (35.3)	

Data are shown as the mean ± SD. ^+^ median (interquartile range). BMI, body mass index; CAP, controlled attenuation parameter; HDL, high-density lipoprotein; HS-CRP, high sensitivity C-reactive protein.

**Table 2:** Parameters associated with NAFLD (CAP ≥ 248 dB/m).

Variables	Odds Ratio	95% Confidence Interval	*p*-Value *
Age, years	1.01	1.00–1.02	0.220
Male	2.30	1.82–2.92	<0.001
Hypertension	1.62	1.26–2.08	<0.001
Diabetes mellitus	1.52	1.07–2.15	0.020
Hypercholesterolemia	1.50	1.16–1.93	0.002
Smoking	1.79	1.33–2.41	<0.001
Body mass index, kg/m^2^	1.29	1.24–1.34	<0.001
BMI ≥ 25, kg/m^2^	5.66	4.36–7.36	<0.001
Fasting glucose, mg/dL	1.02	1.01–1.03	<0.001
Total cholesterol, mg/dL	1.00	1.00–1.00	0.420
Triglyceride, mg/dL ^+^	5.03	3.85–6.58	<0.001
HDL cholesterol, mg/dL	0.96	0.95–0.97	<0.001
HS-CRP (mg/dL)	1.29	0.89–1.87	0.176
Vitamin D ≥ 20 (vs. <20)(ng/mL)	0.75	0.60–0.95	0.014
Vitamin D Quartile			
1st (–14.6)	1 (reference)		0.019 **
2nd (14.7–19.5)	0.82	0.59–1.13	0.218
3rd (19.6–26.7)	0.73	0.53–1.01	0.056
4th (26.8–)	0.60	0.44–0.83	0.002

BMI, body mass index; CAP, controlled attenuation parameter; HDL, high-density lipoprotein; HS-CRP, high sensitivity C-reactive protein. * compared to no NAFLD, ** *p* for trends,^+^ log transformed.

**Table 3:** Multivariate analysis for the association between vitamin D and hepatic steatosis.

Vitamin D	Age, Sex Adjusted		Multivariate Model I		Multivariate Model II	
	OR (95% CI)	*p*-Value	OR (95% CI)	*p*-Value	OR (95% CI)	*p*-Value
≥20 (vs. <20 ng/mL)	0.72 (0.57–0.91)	0.007	0.79 (0.60–1.00)	0.051	0.78 (0.60–1.02)	0.069
Quartile						
1st (–14.6)	1 (reference)	0.006 *	1 (reference)	0.037 *	1 (reference)	0.065 *
2nd (14.7–19.5)	0.73 (0.52–1.02)	0.062	0.70 (0.49–0.99)	0.046	0.68 (0.47–1.00)	0.049
3rd (19.6–26.7)	0.62 (0.45–0.87)	0.005	0.66 (0.46–0.95)	0.024	0.65 (0.44–0.94)	0.022
4th (26.8–)	0.57 (0.41–0.80)	0.001	0.61 (0.43–0.87)	0.007	0.64 (0.44–0.94)	0.021

CI, confidence interval; OR, odds ratio; Model I: Adjusted for age, sex, hypertension, diabetes, hypercholesterolemia and body mass index. Model II: Model I plus high-density lipoprotein cholesterol, triglyceride and smoking. * *p* for trends.

**Table 4:** Multivariate analysis for the association between vitamin D and MAFLD.

Vitamin D	Unadjusted		Age, Sex and Smoking Adjusted		Multivariate Model	
	OR (95% CI)	*p*-Value	OR (95% CI)	*p*-Value	OR (95% CI)	*p*-Value
≥20 (vs. <20 ng/mL)	0.73 (0.59–0.91)	0.005	0.72 (0.57–0.90)	0.005	0.78 (0.59–1.03)	0.079
Quartile						
1st (–14.6)	1 (reference)	0.006 *	1 (reference)	0.015 *	1 (reference)	0.167 *
2nd (14.7–19.5)	0.97 (0.71–1.31)	0.829	0.87 (0.64–1.20)	0.403	0.74 (0.50–1.09)	0.123
3rd (19.6–26.7)	0.85 (0.63–1.16)	0.31	0.74 (0.54–1.02)	0.069	0.69 (0.47–1.03)	0.066
4th (26.8–)	0.60 (0.44–0.82)	0.001	0.60 (0.43–0.83)	0.002	0.67 (0.45–0.99)	0.046

CI, confidence interval; OR, odds ratio; MAFLD, metabolic dysfunction-associated fatty liver disease. Adjusted for age, sex, smoking, waist circumference, systolic blood pressure, triglyceride and high sensitivity C-reactive protein. * *p* for trends.

**Table 5:** Multivariate analysis for the association between grade of hepatic steatosis and vitamin D.

Reference (CAP < 248)	248 ≤ CAP < 302		CAP ≥ 302	
	OR (95% CI)	*p*-Value	OR (95% CI)	*p*-Value
Vitamin D Quartile				
1st (–14.6)	1 (reference)		1 (reference)	
2nd (14.7–19.5)	0.68 (0.45–1.03)	0.069	0.69 (0.42–1.11)	0.127
3rd (19.6–26.7)	0.66 (0.44–0.99)	0.044	0.63 (0.38–1.02)	0.059
4th (26.8–)	0.71 (0.47–1.06)	0.091	0.52 (0.31–0.87)	0.013

CAP, controlled attenuation parameter; CI, confidence interval; OR, odds ratio. Adjusted for age, sex, hypertension, diabetes, hypercholesterolemia, body mass index, high-density lipoprotein cholesterol, triglyceride and smoking.

## Data Availability

The data presented in this study are available on request from the corresponding author.
